# Evaluation of CPP and LDH nanocarriers for dsRNA efficiency in *Brassicogethes aeneus*

**DOI:** 10.3389/finsc.2026.1872725

**Published:** 2026-08-04

**Authors:** Triin Kallavus, Ly Porosk, Liina Soonvald, Apurva Vikas Sabnis, Silva Vilumets, Riina Kaasik, Jonathan Willow, Clauvis Nji Tizi Taning, Kristof De Schutter, Margus Pooga, Eve Veromann

**Affiliations:** 1 Institute of Agricultural and Environmental Sciences, Estonian University of Life Sciences, Tartu, Estonia; 2 Department of Plants and Crops, Faculty of Bioscience Engineering, Ghent University, Ghent, Belgium; 3 Faculty of Science and Technology, Institute of Technology, University of Tartu, Tartu, Estonia; 4 Pheasants Forever and Quail Forever, St. James, MN, United States

**Keywords:** pollen beetle, RNAi, dsRNA, nanoparticle delivery, MgAl-LDH, PF14, pest management

## Abstract

This study evaluates two nanocarrier systems, clay-based magnesium-aluminum layered double hydroxide (MgAl-LDH) and the cell-penetrating peptide PepFect14 (PF14), for delivering double-stranded RNA (dsRNA) targeting the *αCOP* gene in *Brassicogethes aeneus*. Both carriers successfully formed stable complexes and protected dsRNA from degradation under simulated gut conditions. PF14 produced small, uniform nanoparticles (<120 nm), whereas MgAl-LDH generated substantially larger particles (~416 nm), potentially limiting cellular uptake. Feeding assays revealed that naked dsRNA caused high mortality (93% by day 12), while PF14-complexed dsRNA induced substantial mortality (70%) with a delayed onset. In contrast, MgAl-LDH-complexed dsRNA achieved only 22% mortality despite providing strong protection against degradation. mRNA expression analysis at day 3 and 6 showed moderate, statistically insignificant early *αCOP* downregulation, consistent with delayed intracellular processing. At higher concentrations (600 ng/µL), naked dsRNA strongly suppressed *αCOP* transcripts (100%), whereas MgAl-LDH complexes produced only modest knockdown (63.6%). Overall, these findings suggest that while MgAl-LDH offer robust dsRNA stabilization, its delivery efficiency may be constrained by particle size and gut physiology. PF14 demonstrates promise as a carrier enabling sustained delivery with delayed onset, whereas MgAl-LDH requires higher dsRNA doses to achieve comparable effects. Carrier selection should balance dsRNA stability with timely release and account for species-specific gut barriers to optimize RNAi-based pest control strategies.

## Introduction

The rapeseed oil market is experiencing significant growth, driven by diverse industrial applications and environmental considerations. Oilseed rape (*Brassica napus* L.), as a versatile crop, is primarily cultivated for the production of cooking oil, animal feed, and biofuels (1–3). However, its production is significantly constrained by insect pests, particularly the pollen beetle *Brassicogethes aeneus* Fab. (Coleoptera: Nitidulidae), which is a major pest of oilseed rape across Europe. Adult beetles cause substantial yield losses by feeding on young flower buds during the early reproductive stages (4, 5). Crop damage is especially severe when beetle immigration coincides with the green bud stage, which represents the most vulnerable developmental phase of the crop. Pest management in oilseed rape relies predominantly on synthetic insecticides; however, increasing resistance development in *B. aeneus* populations has raised serious concerns regarding the long-term sustainability and effectiveness of these control strategies (6–8). This trend underscores the urgent need for alternative, environmentally friendly pest management approaches.

RNA interference (RNAi) has emerged as a promising species-specific pest control strategy, offering reduced environmental impact, high target specificity, and biodegradability. RNAi functions through sequence-specific degradation of messenger RNA (mRNA), leading to downregulation of essential genes and subsequent impairment of insect survival and fitness (9, 10). Among potential targets, the *αCOP* gene encodes a subunit of the coatomer protein complex I (COPI), which is essential for vesicular transport between the Golgi apparatus and endoplasmic reticulum. Disruption of *αCOP* function interferes with intracellular protein trafficking and membrane dynamics, and has been proposed as a potentially essential target for RNAi-based pest control. The *αCOP* gene has also been assessed for potential effects in non-target organisms in previous studies (10).

Despite its potential, the practical application of dsRNA is limited by rapid degradation in insect digestive systems and inefficient cellular uptake, primarily due to nuclease activity and physiological gut barriers (11, 12). To overcome these limitations, nanocarrier systems have been developed to protect dsRNA and enhance delivery efficiency (13). In addition to widely studied systems such as liposomes and chitosan-based carriers, novel inorganic and peptide-based platforms have gained attention, including magnesium–aluminum layered double hydroxide (MgAl-LDH) and cell-penetrating peptides (CPPs), such as PepFect 14 (PF14) (14).

MgAl-LDH clays have been widely investigated due to their ability to form stable complexes with nucleic acids and protect dsRNA from enzymatic degradation. Acting as reservoirs, these layered materials enable gradual release of dsRNA, thereby prolonging exposure and enhancing RNAi efficacy (15, 16). Their biocompatibility, low toxicity, and high anion-exchange capacity further support their suitability for agricultural applications (17, 18). In contrast, PF14 is an amphipathic lipopeptide capable of forming compact complexes with nucleic acids and facilitating rapid cellular uptake and intracellular delivery, making it an efficient carrier for RNAi applications (19, 20).

CPP-based delivery systems have been shown to enhance RNAi efficiency in various insect species. For example, Vogel et al. (21) demonstrated that CPP–dsRNA complexes can protect dsRNA from gut degradation in *Schistocerca gregaria* Forsskål, although improvements in gene silencing efficiency were variable. Similarly, Wang et al. (22) reported enhanced RNAi efficacy in *Helicoverpa armigera* Hübner through improved cellular uptake mediated by CPPs. These studies indicate that CPPs are promising delivery vehicles, although optimization of peptide design and dosing remains necessary for consistent biological effects.

Despite these advances, the comparative performance of nanocarrier systems in coleopteran pests such as *B. aeneus* remains poorly understood. While coleopterans such *as Leptinotarsa decemlineata* Say are known to be highly RNAi-sensitive (23), other species exhibit variable responses, and the influence of delivery systems on this variability is still under investigation (19, 24). Carrier type, target gene, and insect physiology may all influence the timing and magnitude of gene silencing and mortality outcomes.

In addition, the most current studies rely on artificial feeding systems, such as sucrose-based assays, which allow controlled assessment of delivery efficiency but do not fully represent plant-mediated exposure conditions relevant to agricultural settings. Therefore, understanding nanocarrier performance under simplified laboratory conditions is an important first step toward application in crop protection systems.

This study evaluated the physicochemical properties, dsRNA protection capacity, and biological efficacy of MgAl-LDH and PF14 nanocarriers for delivery of dsRNA targeting *αCOP* in *B. aeneus* under their respective experimental conditions. Specifically, we evaluated their ability to protect dsRNA from degradation, induce gene silencing, and trigger mortality in feeding assays, thereby providing mechanistic insight into how nanocarrier design influences RNAi efficiency in a coleopteran pest of oilseed rape under laboratory conditions.

## Materials and methods

### Insects and dsRNA

Pollen beetles were collected in 2025 from an organic oilseed rape field located near Tartu, Estonia (58.364715°N, 26.666138°E). Species were identified morphologically using the key of Kirk-Spriggs (25) and only *B. aeneus* specimens were used in subsequent experiments. The beetles were kept in a controlled environment chamber (Sanyo MLR-351H, Osaka, Japan) at 20 ± 2 °C, 70% relative humidity, with a photoperiod of 16 h light and 8 h darkness, and were fed *ad libitum* on rapeseed flower pollen.

The dsRNA sequences used in the study were selected based on our previous experiments (10, 26–30). A 455 base-pair dsRNA fragment complementary to a region of the green fluorescent protein (dsGFP) mRNA served as the control treatment. For targeting *B. aeneus* coatomer subunit α (dsαCOP), a 213 bp segment of the coding region was chosen. All dsRNA constructs were synthesized *in vitro* by AgroRNA (Genolution, Seoul, South Korea) and supplied pre-dissolved in dH₂O according to the manufacturer’s standard protocol. Upon arrival, the dsRNA solutions were stored at 5 ± 1 °C. Detailed information regarding the target genes and primer sequences is provided in the Supplementary Information (**SI Tables 1**, **2**).

### Nanocarrier preparation and dsRNA–nanocarrier complex formation

Clay-based nanocarriers were prepared by suspending a commercially obtained magnesium–aluminum layered double hydroxide (MgAl-LDH) slurry (SMT Smallmatek, Portugal) in Milli-Q grade water and sonicating for 3 minutes using a medium-sized probe sonicator (pulse: 1 s on/1 s off, amplitude: 30%) to ensure uniform dispersion.

To form dsRNA–clay complexes, various clay-to-dsRNA mass ratios (0.1% to 1.2%, calculated based on dry mass) were tested. Equal volumes of dsRNA solution and clay suspension (1:1 volume ratio) were gently mixed by pipetting approximately ten times and incubated at room temperature (21 ± 1 °C) for 30 minutes.

Peptide nanocarriers were prepared by reconstituting the PF14 cell-penetrating peptide (CPP) (GL Biochem Ltd, China) to 1 mM using either dry ethanol (for feeding assays) or a solvent mixture of 10% DMSO and 90% dry ethanol. Solvent selection was based on previous studies (20, 31). All solvents were of analytical grade (p.a.) with purity >99.5% and low water content: ethanol (Chem-Lab, Belgium) and DMSO (AppliChem, Germany). Stock solutions were stored at −25 ± 1 °C and equilibrated to room temperature prior to use.

Peptide concentration was verified spectrophotometrically using the extinction coefficient of the tyrosine residue and adjusted as needed. The PF14 sequence was: Stearoyl-AGYLLGKLLOOLAAAALOOLL-NH₂, with a net charge of +5 (based on lysine and ornithine residues), used for calculating charge ratios (CR, N/P).

To form peptide–dsRNA complexes, dsRNA was first added to the reaction vessel, followed by peptide solution to achieve CR values ranging from 1 to 3. The mixture was gently pipetted for at least 10 seconds and incubated at room temperature (22 ± 1 °C) for 30 minutes.

### Physicochemical and functional characterization of nanoparticle complexes

To evaluate the physicochemical properties and stability of dsRNA–nanoparticle complexes, hydrodynamic diameter, polydispersity index (PDI), and zeta potential were measured using a Malvern Zetasizer system (Ultra for clay-based and Nano ZSP for peptide complexes) with ZEN0040 cuvettes. Measurements were performed on freshly prepared complexes diluted in ultrapure water. These parameters provided insight into nanoparticle size distribution, surface charge, and colloidal stability. The accessibility of dsRNA within the clay-dsRNA nanocarrier complexes and CPP-dsRNA nanoparticle was evaluated using the fluorescent nucleic acid intercalating dye, Quant-it™ RiboGreen (Thermo Fisher Scientific) or equivalent. Nanocarrier-dsRNA complexes were prepared as detailed in a previous section (Nanocarrier preparation and dsRNA–nanocarrier complex formation). Following incubation at room temperature, the complexes were diluted to a concentration of 33.3 ng in 10 µL of the complex solution. Subsequently, 10 µL samples were added to a black 96-well plate containing the diluted intercalating dye. After a 10-minute incubation at room temperature, shielded from light, the fluorescence was measured using a SynergyMx spectrophotometer (BioTek) with excitation and emission wavelengths set to 492 nm and 535 nm, respectively. Results were normalized to the fluorescence of free nucleic acids at the same concentration (100%), while fluorescence from ultrapure water, both with and without dye, was utilized to establish the background (0%). The results are expressed as the percentage of fluorescence signal from accessible nucleic acids compared to that of the free nucleic acids.

To assess the stability of these nanocarrier-dsRNA complexes, we examined their response to the polyanion heparin, which can strip positively charged components from complexes, thereby enhancing the accessibility of nucleic acids for the dye. The incubation involved adding either 10 mg/mL or 50 mg/mL heparin solution in a 1:1 (v:v) ratio to the complex solutions, achieving final concentrations of 5 mg/mL and 25 mg/mL respectively. This mixture was then incubated at 37 °C for 30 minutes with gentle shaking. After incubation, the samples were diluted with water to maintain a concentration of 33.3 ng in 10 µL of complex solution, followed by transfer to a black 96-well plate that contained 90 µL of the diluted intercalating dye.

Additionally, clay-based complexes (85 μL) were subjected to surfactant-induced decomplexation by adding 30 μL of 1% sodium dodecyl sulfate (SDS) solution and incubating for 15 minutes at room temperature. Released dsRNA was analyzed via agarose gel electrophoresis (1.5% agarose in 0.5× TAE buffer, 100 V, 30 min), stained with ethidium bromide (0.5 mg/L), and visualized using a GelDoc XR+ imaging system (Bio-Rad).

### Enzymatic degradation assay using *Brassicogethes aeneus* gut extract

To assess the stability of dsRNA delivered via different nanocarriers, two systems, peptide- and clay-based carriers, were evaluated for their ability to protect dsRNA from degradation under simulated insect gut conditions. To ensure consistency across experiments, the preparation of gut extracts, incubation procedures, and analytical methods were standardized.

Gut fluid was obtained by dissecting four adult *B. aeneus* guts and transferring them into 100 µL of phosphate-buffered saline (PBS) without homogenization. Samples were kept on ice prior to centrifugation at 20,000 × g for 10 minutes, and the supernatant was collected for further analysis. Total protein concentration of gut extracts was determined using a Bradford assay with Coomassie Brilliant Blue reagent. Briefly, 10 µL of sample was mixed with 100 µL of reagent, and absorbance was measured at 595 nm. PBS with reagent served as a blank. A standard curve was generated using bovine serum albumin (BSA), and protein concentrations were calculated accordingly. Gut extracts contained approximately 18 µg/mL total protein.

Following centrifugation, 35 µL of the supernatant was combined with 50 µL of nanocarrier–dsRNA complex. The dsRNA concentration differed between carrier systems due to formulation-specific optimization and biological relevance in feeding assays. Therefore, results should be interpreted within each system rather than as direct cross-carrier comparisons. This approach allows interpretation of stability under application-relevant conditions.

Incubation times with gut extract ranged from 15, 30, 60, to 120 minutes depending on the stability profile being investigated. Naked dsRNA incubated under identical conditions served as a control.

To evaluate dsRNA integrity, all samples were treated with 30 µL of 1% sodium dodecyl sulfate (SDS) and incubated for 15 minutes at room temperature. SDS was used to dissociate nanocarrier complexes and inhibit nuclease activity prior to electrophoretic analysis. dsRNA integrity was subsequently assessed via agarose gel electrophoresis.

For peptide carriers, charge ratios (CR) of 2 and 3 were tested using dsαCOP at a concentration of 60 ng/µL. Each condition was run in duplicate across five time points: 0, 15, 30, 60, and 120 minutes. For clay-based carriers, stability was assessed at clay-to-dsRNA ratios of 1.2% and 0.8% (dry mass basis), using dsRNA concentrations of 600 ng/µL, 300 ng/µL, and 60 ng/µL. Each condition was tested across four time points: 0, 15, 30, and 60 minutes.

### Feeding experiments

To evaluate the efficiency of different nanocarriers in delivering dsRNA to insects, feeding experiments were conducted using a suspension of nanoparticles in a 25% sucrose solution under chronic exposure conditions (12 days). The dsRNA was complexed with either a clay-based carrier or a peptide nanocarrier (PF14). Treatments and applied concentrations are presented in **Table 1**.

**Table 1:** Details of treatments used in *Brassicogethes aeneus* feeding experiments.

Feeding method	Nanocarrier type	Treatments
Sugar water (25%)	Clay nanocarrier	Sugar water (25%). Clay 0.8%. dsGFP (60 ng/µL). dsαCOP (60 ng/µL). dsαCOP (300 ng/µL). dsαCOP (600 ng/µL). dsαCOP (60 ng/µL) + clay 0.8%. dsαCOP (300 ng/µL) + clay 0.8%. 9. dsαCOP (600 ng/µL) + clay 0.8%.
Sugar water (25%)	Peptide nanocarrier (PF14)	Sugar water (25%). dsGFP (60 ng/µL). dsαCOP (60 ng/µL). dsGFP (60 ng/µL) + PF14. dsαCOP (60 ng/µL) + PF14.

For clay-based treatments, carrier-only controls were included to evaluate potential effects of the material itself in the absence of dsRNA. For peptide-based treatments, dsGFP–PF14 was used as a non-specific control to account for potential effects of both the carrier and formulation matrix. PF14 alone was not included in survival assays due to practical constraints (including cost and experimental capacity). In addition, only a single dsRNA concentration (60 ng/µL dsαCOP) was tested for peptide-based formulations because of the high cost of peptide synthesis and limited experimental capacity. In contrast, clay-based formulations were evaluated at multiple dsRNA concentrations (600, 300, and 60 ng/µL dsαCOP) to assess dose-dependent effects.

Prior to exposure, adult beetles were starved for 24 hours to synchronize feeding activity. Insects were then placed into transparent, ventilated polystyrene rearing dishes (10 cm diameter × 4 cm height; SPL Life Sciences, Gyeonggi-do, South Korea), with eight randomly selected individuals per dish. The dsRNA–nanocarrier complexes in 25% sucrose solution were administered in modified caps of 1.5 mL Eppendorf tubes, each containing 100 µL of treatment solution.

Feeding assays were conducted separately for each nanocarrier type but under identical environmental and handling conditions to minimize systematic bias. Each treatment was replicated five times per experimental run with eight insects per replicate (in total 40 beetles), and the experiment was repeated across three independent experimental runs (biological replicates; n = 3). In total, 120 insects were tested per treatment group.

Insect survival was monitored daily over a 12-day period. Mortality observed on the first day was excluded from statistical analysis, as it was attributed to handling stress and short-term environmental disturbance rather than treatment-related effects. All survival data were therefore analyzed using corrected mortality, which accounts for background mortality in control groups and allows comparison between independent experimental runs with slight variation in baseline survival.

Relative mRNA expression levels were measured using quantitative PCR (qPCR) at two time points: days 3 and 6 post-exposure for clay-based treatments, and days 2 and 5 for peptide treatments. The time points were selected based on previous studies reporting differences in uptake and RNAi kinetics between delivery systems, and may not fully represent optimal temporal dynamics in *B. aeneus*. (27, 30, 32). Early sampling for PF14 treatments was used to account for potentially faster cellular uptake and earlier onset of gene silencing (33).

For each treatment, the eight insects were pooled into a single 1.5 mL Eppendorf tube to constitute one biological replicate. Three independent biological replicates were prepared per treatment, collected at different times. Each biological replicate was analyzed in technical triplicate by qPCR to ensure measurement reliability.

### qPCR analyses

Insects used in qPCR analysis were homogenized in 600 µL of RLT (RNA Lysis Tissue buffer; with 10 µL of β-mercaptoethanol added per 100 µL), using sterilized plastic pestles and Eppendorf tubes. The homogenates were stored at –80 °C until further processing. Total RNA was extracted using the RNeasy Mini Kit (Qiagen, Venlo, The Netherlands), and RNA concentration and purity were determined using a Nanodrop spectrophotometer (Thermo Scientific, Wilmington, USA). RNA integrity was additionally verified by agarose gel electrophoresis. To eliminate genomic DNA contamination, samples were treated with the Turbo DNA-Free Kit (Invitrogen, Carlsbad, USA) according to the manufacturer’s instructions.

Complementary DNA (cDNA) was synthesized from 1 µg of total RNA using the FIREScript RT cDNA Synthesis Kit (Solis BioDyne, Tartu, Estonia). qPCR was performed using a QuantStudio 3 Real-Time PCR System (Applied Biosystems, Foster City, USA). Each 20 µL reaction contained 4 µL of 5× HOT FIREPol EvaGreen qPCR Supermix (Solis BioDyne), 0.5 µL each of 10 µM forward and reverse primers (Microsynth, Balgach, Switzerland; see **Supporting Information Table 1**), 14 µL of PCR-grade water, and 500 ng of cDNA. The thermal cycling conditions included an initial denaturation at 95 °C for 15 minutes, followed by 37 cycles of 95 °C for 15 seconds and 59 °C for 1 minute. A melting curve analysis from 60 °C to 95 °C was performed at the end of each run to confirm amplification specificity.

### Data analyses

Mortality data from the nanoparticle feeding experiments were analyzed by comparing all treatment groups (naked dsRNA and nanocarrier–dsRNA complexes) with the dsGFP control group, which served as the primary reference for statistical testing of RNAi-specific effects. Background mortality was accounted for using Abbott’s correction formula, based on the sucrose-only control group (25% sucrose solution), which represented baseline survival under experimental conditions. This approach ensured that mortality estimates were adjusted for natural or handling-related death, while statistical comparisons between treatments were performed relative to the dsGFP control. Prior to statistical testing, the assumptions of homogeneity of variances and normality of data distribution were evaluated using Levene’s test and the Shapiro–Wilk test, respectively. As the distribution of data was not normal, the nonparametric Kruskal–Wallis test was employed as an alternative to ANOVA. *Post-hoc* pairwise comparisons were conducted using the Wilcoxon rank-sum test with Bonferroni correction.

For mRNA expression analysis in the nanocarrier experiments, all concentrations of both naked and complexed dsRNA treatments were compared. Relative mRNA expression levels of *αCOP* were normalized using the reference genes *rps3* and *actin*, with normalization and stability assessment automatically performed in qBase, which accounts for differences in reference gene expression across samples. Changes in mRNA expression were calculated as fold change (FC) relative to the control group, and log₂-transformed fold changes (log₂FC) were used for statistical analyses to normalize the data and allow symmetric interpretation of gene up- and downregulation. Cohen’s d was calculated as the standardized mean difference between treatment and control groups. Statistical evaluation of mRNA silencing effects was performed using one-way ANOVA followed by Tukey’s HSD *post-hoc* test for pairwise comparisons between treatment groups. To ensure consistency across experiments, all results are presented as comparisons between dsRNA alone and dsRNA combined with nanocarriers.

All statistical analyses related to physicochemical and functional characterization of nanoparticle complexes (2-way ANOVA) were performed with Statistica software (TIBCO Software Inc., Palo Alto, CA, USA) (2020 Data Science Workbench, version 14). Graph compilation and analysis was performed using GraphPad Prism version 9.5.1 for Windows (GraphPad Software, San Diego, CA, USA). Statistical significance was determined using one-way ANOVA followed by Dunnett’s multiple comparison test. For accessibility assays, free dsRNA served as the reference group, and for heparin displacement assays, the corresponding water-treated samples were used as controls.

All statistical analyses related to mortality were performed using R version 3.6.3 (R Foundation for Statistical Computing, Vienna, Austria), with the following R packages: tidy verse (34), tidyr (35), dplyr (36), knitr (37), broom (38), statix (39), Rmisc (40), multcomp (41), readxl (42), ggplot2 (43), and forcats (44).

## Results

### Nanocarrier complexation, characteristics and functional assays

The clay-based nanocarrier MgAl-LDH efficiently complexed dsRNA at clay-to-dsRNA ratios above 0.3% (based on dry mass), with native gel electrophoresis confirming complete binding at these concentrations. However, formulations exceeding 1.2% clay could not be dissociated using 30 µL of 1% SDS (final concentration 0.26%), indicating excessively strong clay-dsRNA interactions (**SI Figure 1**). Based on gel results and formulation consistency, the 0.8% dry mass formulation was selected for further experiments. This formulation produced particles with an average diameter of 416.1 nm and a polydispersity index (PDI) of 0.3, consistent with previously reported values for MgAl-LDH particles (SMT Smallmatek, Portugal). Zeta potential measurements revealed a charge shift across formulations: 0.4% clay complexes exhibited a negative surface charge, whereas 0.8% and 1.2% clay complexes were positively charged, reflecting higher clay-to-dsRNA ratios and stronger electrostatic interactions (**SI Figure 2A**).

RiboGreen assay confirmed effective dsRNA encapsulation, with fluorescence intensity increasing progressively as clay concentration increased (**SI Figure 2B**). Although the lowest signal was observed at 0.4%, the 0.8% formulation was chosen for further use due to its consistent performance across assays. Heparin displacement assays showed a concentration-dependent response: at 5 mg/mL heparin, the 0.4% formulation released less than 25% of dsRNA, while 0.8% and 1.2% formulations released over 50%. At 25 mg/mL, both 0.8% and 1.2% formulations released more than 75% of dsRNA, whereas the 0.4% formulation remained comparatively more stable, releasing less than 50% (**SI Figure 2C**).

Stability assays using insect gut extract further demonstrated the protective capacity of clay-based complexes, with intact RNA detectable up to 60 minutes under simulated gut conditions (**SI Figure 3**).

In contrast, PF14 nanocarriers were evaluated at charge ratios CR 2 and CR 3 for their ability to condense dsRNA into nanoparticles. Both formulations yielded particles smaller than 120 nm with PDI values ≤ 0.4. Specifically, CR 2 produced particles with an average size of 118.6 nm and a PDI of 0.4, while CR 3 resulted in smaller and more uniform particles (87.5 nm, PDI 0.2; **SI Figure 4A**). Zeta potential measurements revealed a similar charge shift: CR 2 complexes exhibited a negative surface charge, whereas CR 3 complexes were positively charged, reflecting increased peptide-to-dsRNA ratio and stronger electrostatic interactions (**SI Figure 4B**).

RiboGreen assay confirmed successful complex formation, with fluorescence significantly reduced compared to free dsRNA controls. Among PF14 formulations, CR 3 showed the lowest fluorescence signal, suggesting the highest degree of dsRNA shielding (**SI Figure 4C**). Nevertheless, CR 2 was selected for subsequent experiments to balance encapsulation efficiency with formulation stability and biological compatibility. Heparin displacement assays showed that at 5 mg/mL heparin, PF14 complexes at CR 2, CR 3, and CR 4 retained more than 50% of bound dsRNA, indicating relatively strong electrostatic interactions (**SI Figure 4D**). However, at 25 mg/mL, all three formulations released over 75% of dsRNA, demonstrating susceptibility to displacement under highly competitive pressure. PF14-based complexes also provided measurable protection in insect gut extract, preserving dsRNA integrity under the tested conditions (**SI Figure 5**), supporting their potential for RNA delivery applications.

### *Brassicogethes aeneus* mortality and mRNA expression – feeding assay with nanocarrier-complexed and naked dsRNA

#### Clay-based nanocarrier

**Figure 1:**
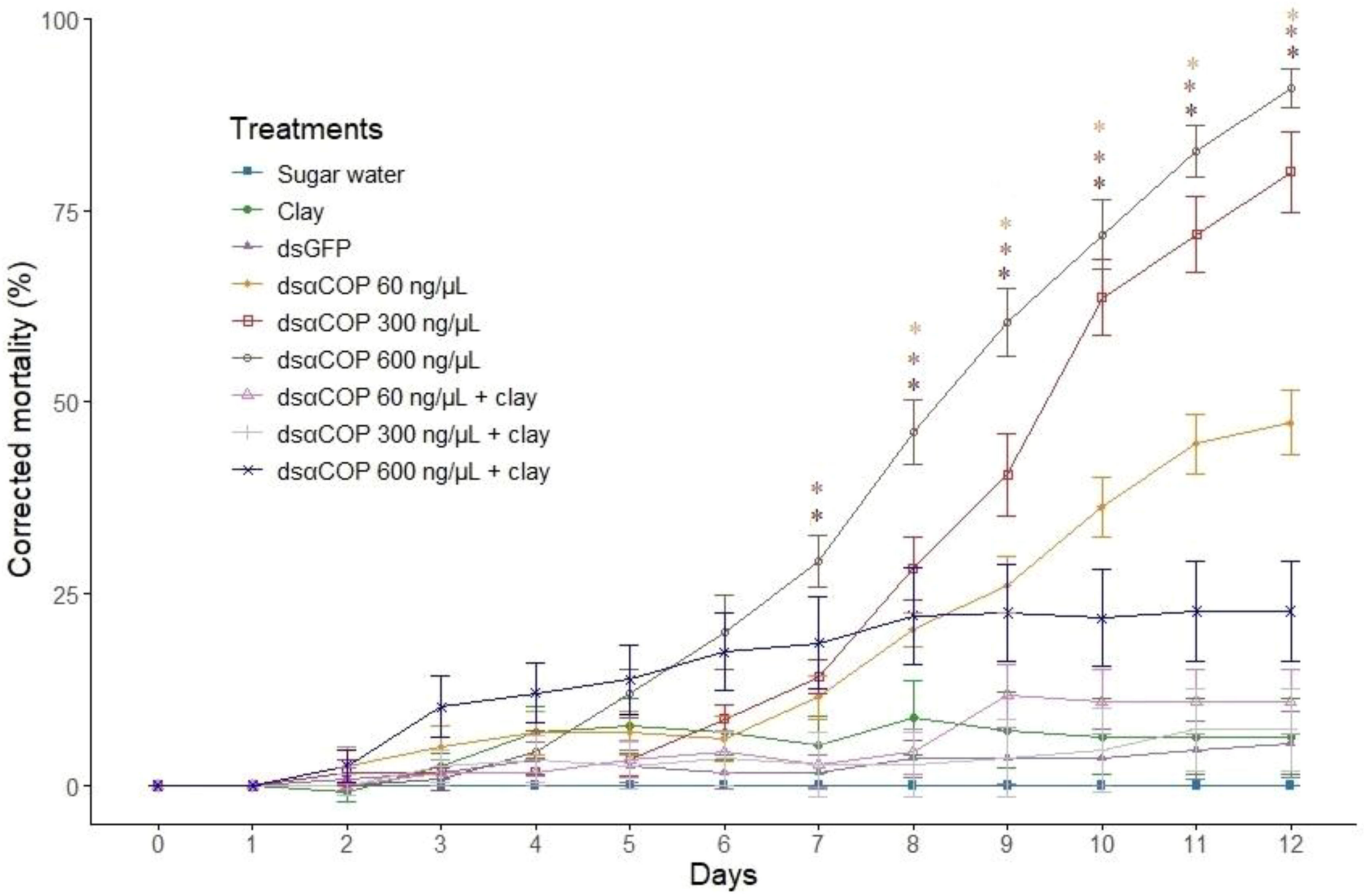
Corrected mortality (%) of *Brassicogethes aeneus* adults fed with dsαCOP (60 ng/µL, 300 ng/µL, 600 ng/µL), each with and without the clay nanocarrier, across three experimental replicates. Mortality values were corrected using the sugar water control. Asterisks (*) denote statistically significant differences between dsαCOP treatments and the dsGFP control (df = 8, p < 0.05, Kruskal-Wallis test followed by Wilcoxon rank-sum test with Bonferroni correction [Bonferroni correction factor (α): 0.001388889)]. The color of the asterisk corresponds to the respective treatment group. Error bars represent ± SEM.

**Figure 2:**
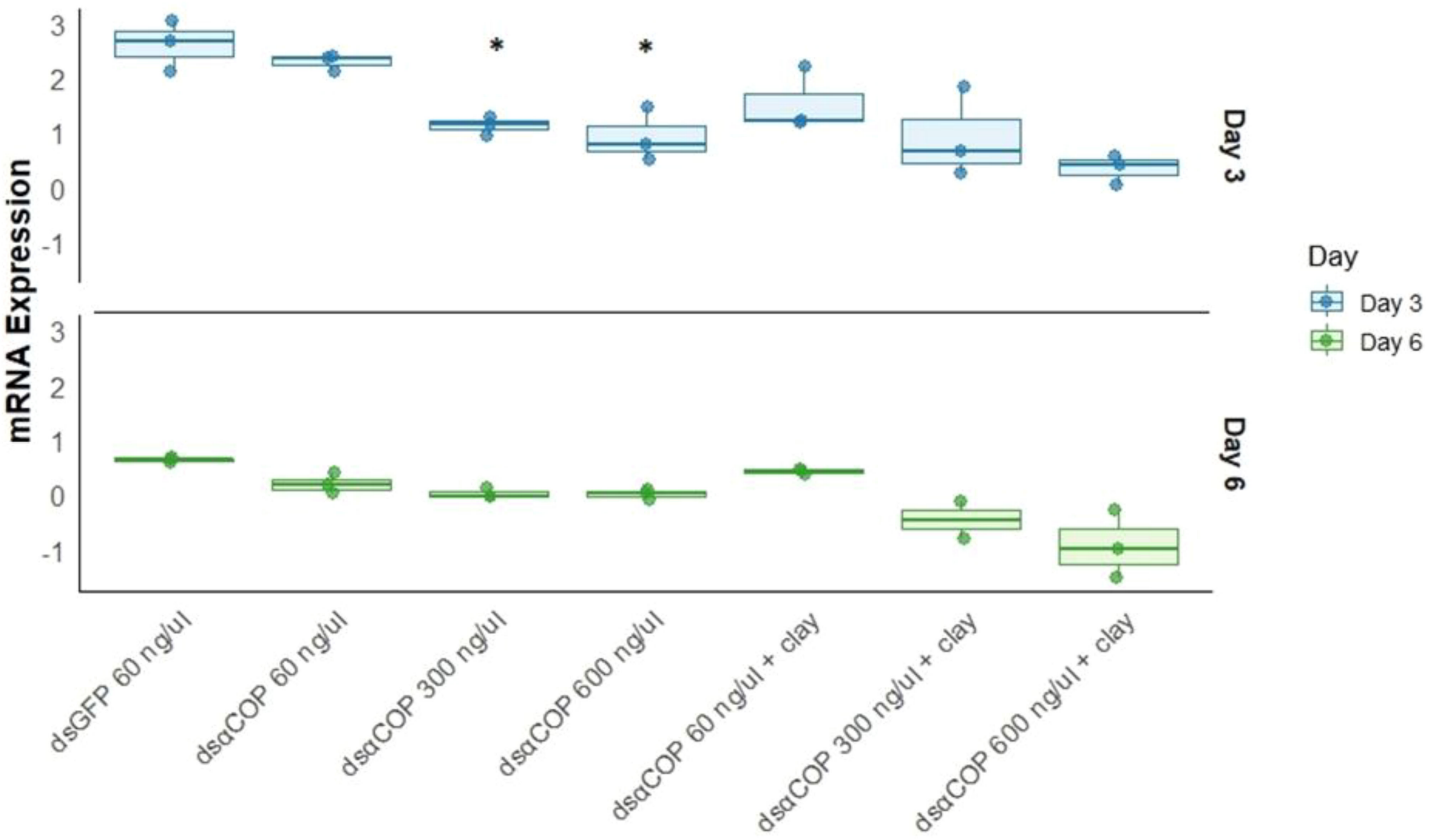
Relative *aCOP* mRNA expression after dietary exposure to dsaCOP treatments with and without clay, in *Brassicogethes aeneus* sampled at 3 and 6 days (n = 3 biologically independent qPCR samples per treatment and time point). Relative GFP mRNA expression was assessed using qBase without manual setting of calibration groups. Normalization was performed using the reference genes *rps3* and *actin*, and relative mRNA values were calculated automatically by qBase. Therefore, control group values are shown as computed by qBase and may differ from 1.0. Error bars represent the standard error of the mean (SEM) from biological replicates. Asterisks indicate significant differences of dsαCOP treatment compared to dsGFP controls (p < 0.05; one-way ANOVA). All data are presented on the same scale for comparison.

Exposure to sugar water containing dsαCOP at a concentration of 600 ng/µL resulted in a pronounced increase in *B. aeneus* adult mortality, reaching 29.2% by day 7 (df = 8, p < 0.05) and rising sharply thereafter to approximately 90% by day 12 (df = 8, p < 0.001; **Figure 1**). These results highlight the strong RNAi-mediated lethality associated with *αCOP* silencing at this concentration when delivered in a simple sugar solution (**Figure 2**).

In contrast, when dsαCOP (600 ng/µL) was administered in complex with the MgAl-LDH clay, mortality was considerably lower. By day 9, mortality reached 22% (df = 8, p > 0.05), and remained unchanged through day 12, indicating a plateau in efficacy (**Figure 1**).

mRNA expression analysis on day 3 revealed statistically significant downregulation of the *αCOP* target gene in naked dsRNA treatments compared to the dsGFP control (**Figure 2**). Beetles treated with 300 ng/µL dsαCOP showed a log₂ fold change of -1.21 (56.8%, Cohen’s d = 3.4, p < 0.05), while those exposed to 600 ng/µL exhibited even stronger suppression, with a log₂ fold change of -1.50 (64.7%, Cohen’s d = 2.8, p < 0.05), confirming a dose-dependent RNAi response. The 60 ng/µL dsαCOP treatment did not significantly affect target mRNA levels (log₂ fold change of -0.19 (12.3%, Cohen’s d = 0.73, p > 0.05). dsαCOP-clay complexes delivered at 600 ng/µL produced statistically not-significant downregulation (log₂ fold change = -1.46, 63.6%, Cohen’s d = 1.15, p > 0.05), similar to the silencing observed in the naked dsRNA treatment at the same concentration. This suggests that clay-mediated delivery achieved measurable mRNA downregulation, but this did not translate into proportional mortality under the tested conditions, indicating a dissociation between transcript suppression and organismal outcome.

By day 6, transcript suppression became more pronounced, particularly in the dsRNA treatments without nanoparticle. Compared to the dsGFP control, the 60 ng/µL treatment resulted in a log₂ fold change of -1.73 (69.8%, Cohen’s d = 2.69), though not statistically significant (p > 0.05). In contrast, the 300 ng/µL and 600 ng/µL treatments led to dramatic reductions in *αCOP* expression, with log₂ fold changes of -5.66 (98.0%, Cohen’s d = 7.13) and -13.3 (100.0%, Cohen’s d = 7.03), respectively (p > 0.05).

#### Peptide nanocarrier

**Figure 3:**
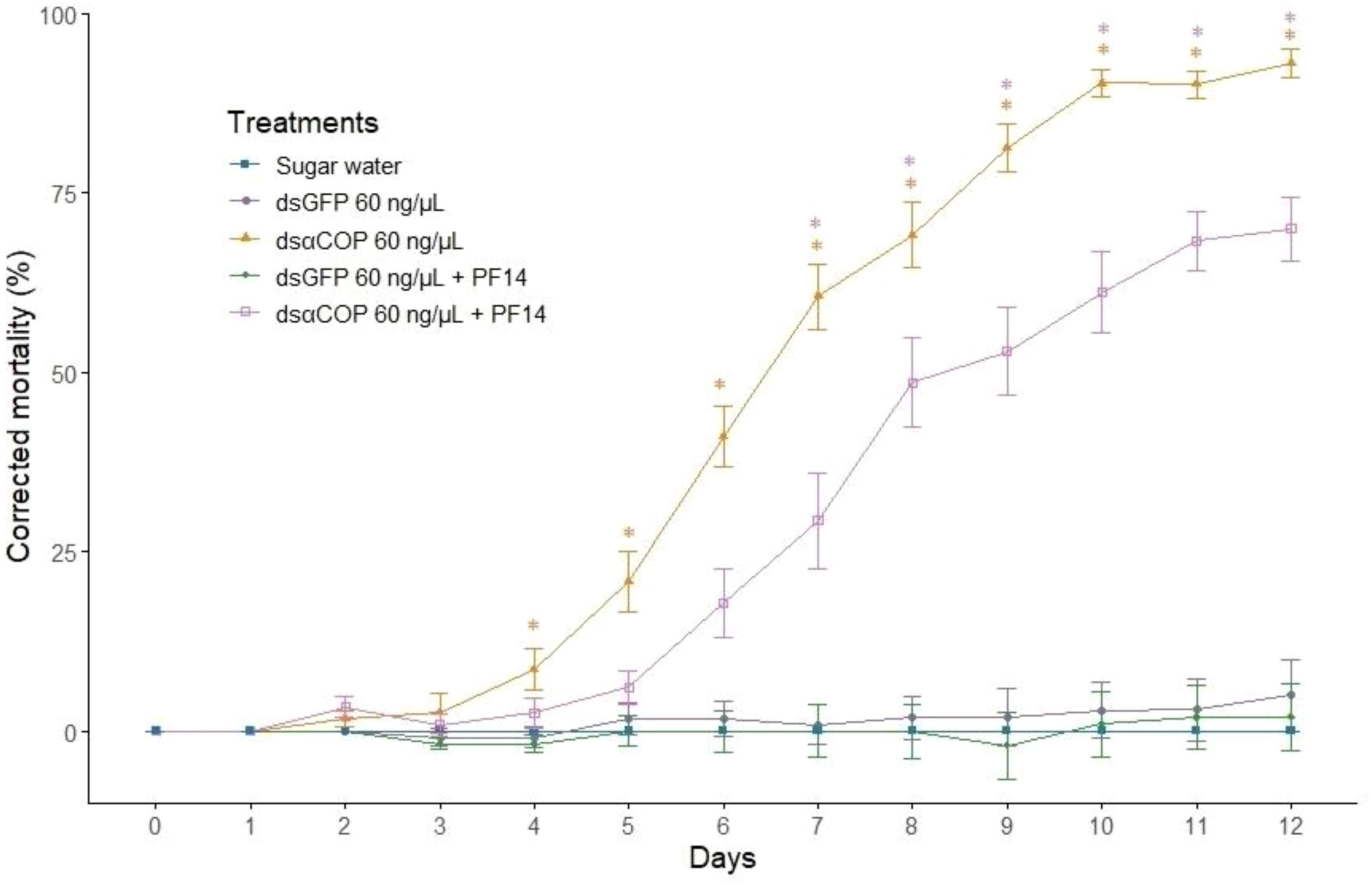
The mean (± SEM) percentages of corrected mortality (%) of *Brassicogethes aeneus* adults fed with dsαCOP (60 ng/µL), each with and without the peptide PF14, across three experimental replicates. Mortality values were corrected using the sugar water control. Asterisks (*) denote statistically significant differences between dsαCOP treatments with the dsGFP control (df = 4, p < 0.05, Kruskal-Wallis test followed by Wilcoxon rank-sum test with Bonferroni correction [Bonferroni correction factor (α): 0.005)]. The color of the asterisk corresponds to the respective treatment group.

A significant increase in mortality was observed in *B. aeneus* adults following dietary exposure to dsαCOP delivered in sugar water. Specifically, when beetles were fed with a 60 ng/µL concentration of dsαCOP in sugar solution, corrected mortality reached approximately 90% by the end of the 12-day observation period (**Figure 3**). Notably, mortality was already evident by day 6, with 40% of individuals deceased (df = 4, p < 0.0001), and continued to rise sharply, culminating in 93% mortality by day 12 (df = 4, p < 0.0001). These results indicate that dsαCOP alone can induce strong RNAi effects under sucrose-feeding conditions, supporting *αCOP* as a functionally essential target in this species.

When dsαCOP (60 ng/µL) was complexed with the cell-penetrating peptide PF14, the mortality effect was delayed. On day 7, only 29% mortality was recorded (df = 4, p < 0.05); however, by day 12, mortality had risen to 70% (df = 4, p < 0.0001), indicating that PF14-mediated delivery resulted in delayed but substantial lethality over the observation period.

**Figure 4:**
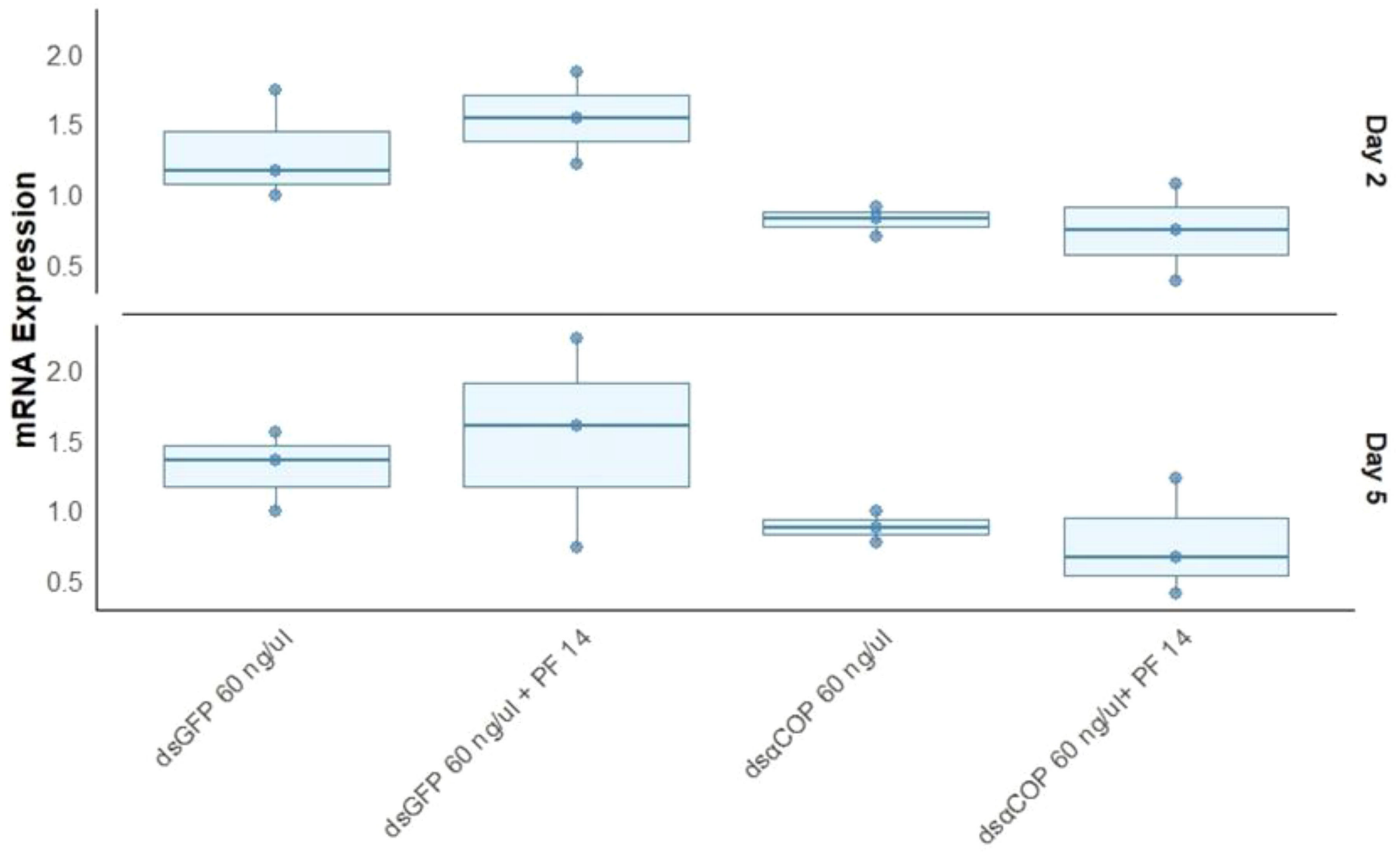
Relative *aCOP* mRNA expression after dietary exposure to dsaCOP treatments with and without peptide, in *Brassicogethes aeneus* sampled at 2 and 5 days (n = 3 biologically independent qPCR samples per treatment and time point). Relative GFP mRNA expression was assessed using qBase without manual setting of calibration groups. Normalization was performed using the reference genes *rps3* and *actin*, and relative mRNA values were calculated automatically by qBase. Therefore, control group values are shown as computed by qBase and may differ from 1.0. Error bars represent the standard error of the mean (SEM) from biological replicates. Asterisks indicate significant differences of dsαCOP treatment compared to dsGFP controls (p < 0.05; one-way ANOVA). All data are presented on the same scale for comparison.

mRNA expression analysis mirrored the mortality pattern. On day 2, treatment with naked dsαCOP at 60 ng/µL led to a log₂ fold change of -0.682 (37.3%, Cohen’s d = 1.34) compared to the dsGFP control, indicating a moderate non-significant reduction in transcript levels (p > 0.05) (**Figure 4**). PF14-complexed dsαCOP showed minimal reduction of transcript levels compared to the naked dsαCOP treatment (log₂ fold change = -0.150, 9.8%, Cohen’s d = 0.24, p > 0.05), suggesting delayed onset of RNAi activity.

By day 5, naked dsαCOP 60 ng/µL treatment continued to show modest downregulation of *αCOP* (log₂ fold change = -0.570, 32.6%, Cohen’s d = 1.54, p > 0.05) relative to the dsGFP control, while PF14-complexed dsRNA only remained marginally different from the naked dsRNA (log₂ fold change = -0.201, 13.0%, Cohen’s d = 0.29, p > 0.05), reinforcing the observation that PF14 did not substantially enhance mRNA downregulation under the tested conditions.

## Discussion

This study evaluated the performance of two nanocarrier systems, the cell-penetrating peptide PF14 and the clay-based MgAl-LDH, for delivering dsRNA targeting the *αCOP* gene in *B. aeneus*. Physicochemical characterization confirmed that both carriers formed stable complexes with dsRNA, but PF14 generated small, homogeneous nanoparticles (<120 nm, PI ≤ 0.4), whereas MgAl-LDH formed substantially larger particles (~416 nm, PI = 0.3). These size differences are biologically relevant, as nanoparticles below ~200 nm are generally associated with more efficient cellular uptake via clathrin- or caveolin-mediated endocytosis, whereas larger particles may rely on slower or less efficient uptake routes such as micropinocytosis or phagocytosis (45).

RiboGreen assays confirmed effective dsRNA binding by both carriers, although the binding mode differed. PF14 formed compact complexes within the typical size range reported for CPP-based delivery systems, consistent with amphipathic peptide–nucleic acid interactions that support cellular internalization and intracellular release. In contrast, MgAl-LDH complexes showed greater heterogeneity, consistent with platelet stacking during dsRNA adsorption (46). While this structural tendency may enhance dsRNA stability, it could also limit diffusion through the gut lumen or interaction with epithelial surfaces. Heparin displacement assays further illustrated these mechanistic differences: PF14 retained dsRNA under moderate competitive conditions, suggesting tighter association and more controlled release, whereas LDH complexes released dsRNA in a concentration-dependent manner, consistent with ion-exchange-driven release mechanisms described for LDH systems. Stability assays indicated that both carriers reduced dsRNA degradation under simulated gut conditions compared to naked dsRNA. PF14 preserved dsRNA integrity under simulated gut conditions, and MgAl-LDH extended dsRNA survival in gut extract for up to 60 minutes. These findings are consistent with previous reports on CPP- and LDH-based protection mechanisms (13, 47, 48). However, extracellular stability alone does not ensure effective RNA interference. Functional RNAi requires successful navigation of multiple sequential barriers, including passage through the gut environment, cellular uptake, endosomal escape, cytoplasmic decomplexation, RISC loading, and sustained interaction with target transcripts (49). Limitations at any of these steps may reduce or delay phenotypic responses. The gut environment of *B. aeneus* likely imposes several such constraints that interact differently with each carrier system. The peritrophic matrix, digestive enzymes, and mildly acidic pH may degrade, immobilize, or delay dsRNA or nanocomplexes before they reach epithelial cells. Two major peritrophic matrix types have been described: the dynamic Type I and the more rigid Type II, which differ substantially in permeability and nanoparticle interactions (50–53). These physiological factors likely contribute to the observed differences in delivery efficiency between carriers. (54, 55), these barriers could disproportionately affect larger LDH-based complexes. Although beetles such as chrysomelids are generally considered RNAi-responsive, species-specific variation in gut physiology can markedly influence delivery efficiency and phenotypic outcomes (19, 56). Further characterization of the *B. aeneus* gut environment will therefore be important for refining delivery strategies.

Feeding assays revealed clear differences in phenotypic responses among treatments. Naked dsRNA induced rapid and high mortality (>90% by day 12 at 60 ng/µL), whereas PF14-complexed dsRNA produced a substantial but delayed mortality response (~70%). Importantly, differences in onset and magnitude should be interpreted in the context of delivery kinetics rather than uptake alone. Naked dsRNA typically enters cells inefficiently and lacks inherent endosomal escape capacity (57), yet at sufficiently high concentrations, even limited cytoplasmic entry can result in rapid transcript suppression. In contrast, PF14-mediated delivery may involve additional intracellular trafficking steps, including endosomal processing and cytoplasmic release, which can delay observable phenotypic effects. Similar delays following nanocarrier-mediated dsRNA delivery have been reported in *Spodoptera exigua* and *Schistocerca gregaria* (21, 58, 59), supporting the idea that enhanced stability and delivery control may come at the cost of slower RNAi onset. Although PF14 was not tested as a standalone treatment in this study, results obtained with the dsGFP control indicate that it does not exhibit inherent toxicity. The dsGFP–PF14 treatment did not result in increased mortality compared to controls, suggesting that the observed biological effects are attributable to the targeted dsRNA rather than the carrier itself. These findings support the assumption that PF14 is a biologically compatible delivery system under the conditions tested.

Environmental instability remains a major limitation for RNAi-based pest control. While naked dsRNA can be highly effective under controlled laboratory conditions, it degrades rapidly under UV exposure, temperature fluctuations, and nuclease-rich environments, leading to substantial loss of activity under field-relevant conditions (49, 60). Nanocarriers such as CPPs, LDH, liposomes, and polymers address these challenges by extending dsRNA persistence, facilitating controlled release, and improving uptake, particularly in less RNAi-sensitive systems (19, 21). Thus, delivery efficiency should be evaluated not only by short-term mortality but also by stability, robustness, and suitability for real-world application.

A notable observation in this study was that high concentrations of MgAl-LDH–dsRNA strongly reduced *αCOP* transcript levels (log₂FC −1.46), comparable to or even exceeding the knockdown achieved with naked dsRNA, yet mortality remained relatively low (~22%). This apparent discrepancy suggests that whole-body transcript measurements may not accurately reflect functionally relevant silencing in critical tissues. One plausible explanation is that LDH-mediated delivery leads to uneven tissue distribution and release dynamics, resulting in effective uptake and silencing in some tissues but limited delivery to organs essential for survival, such as the gut or nervous system. Additionally, the timing and persistence of silencing may differ between treatments. LDH-mediated delivery, which relies on ion-exchange-driven release, may result in slower or spatially restricted dsRNA availability compared to naked dsRNA, potentially delaying or weakening downstream physiological effects. It is also important to consider that mRNA reduction does not directly equate to functional protein depletion, as protein turnover rates and compensatory physiological responses can buffer the impact of transcript knockdown. However, this remains a hypothesis and requires validation through tissue-specific expression or protein-level analysis. Future studies should therefore focus on tissue-specific expression analysis, protein-level quantification, and temporal profiling of gene silencing to better resolve the relationship between transcript reduction and phenotypic outcome. Such approaches would help clarify whether the observed decoupling between gene knockdown and mortality reflects limitations in delivery, release kinetics, or downstream biological responses. LDH-based systems have enhanced RNAi responses in some insect species (15), but their performance is highly context-dependent. Particle size, surface charge, release kinetics, and gut physiology collectively determine biological outcomes. For example, Arjunan et al. (13) showed that LDH particle size influences midgut internalization, while Cheng et al. (61) demonstrated that release kinetics affect both the timing and magnitude of mRNA downregulation. In *B. aeneus*, LDH particles may achieve sufficient uptake to reduce transcript levels but may not deliver dsRNA to critical tissues or at the appropriate developmental window to induce mortality.

Overall, these results suggest a potential trade-off in nanocarrier-mediated RNAi between dsRNA protection and the timing and localization of intracellular release under the conditions tested. PF14 appears to support more efficient functional delivery at moderate concentrations, suggesting that further optimization of carrier composition or release kinetics could enhance efficacy. MgAl-LDH provides strong extracellular protection but appears less effective at translating transcript knockdown into phenotypic effects under the conditions tested. For species-specific applications such as *B. aeneus*, carrier selection should therefore consider not only stability and uptake but also tissue targeting, temporal dynamics of silencing, and intrinsic physiological barriers. These insights contribute to the development of mechanistically informed and species-tailored RNAi delivery strategies for pest management.

## Conclusions

This study evaluated the performance of the cell-penetrating peptide PF14 as a dsRNA delivery system for RNA interference targeting the *αCOP* gene in *Brassicogethes aeneus*. PF14 was shown to efficiently protect dsRNA from degradation under the tested conditions and to produce biologically relevant effects in feeding assays. However, PF14 did not result in a consistent or statistically significant enhancement of *αCOP* transcript silencing at early time points (days 2 and 3), regardless of whether dsRNA was delivered naked or in complex with the peptide. At higher dsRNA concentrations, a reduction in transcript levels was observed, indicating that RNAi activity is concentration-dependent under the experimental conditions used. Despite the limited early transcript knockdown, PF14-treated groups showed delayed but substantial mortality over time, suggesting that gene silencing effects may develop after prolonged exposure. This temporal pattern may reflect differences in dsRNA availability and intracellular processing between delivery systems, although the exact mechanisms cannot be resolved based on the present data.

Overall, the results suggest that while PF14 improves dsRNA stability and enables biological activity *in vivo*, its impact on the timing and efficiency of gene silencing differs from naked dsRNA delivery. Rather than providing a straightforward improvement in RNAi efficacy, PF14 appears to modify the kinetics of the response.

## Data Availability

The datasets presented in this study can be found in online repositories. The names of the repository/repositories and accession number(s) can be found in the article/**Supplementary Material**.
