# Supplementary material for: Evaluation of CPP and LDH nanocarriers for dsRNA efficiency in Brassicogethes aeneus
Source: Front Insect Sci. 2026 Aug 4;6:1872725. doi: 10.3389/finsc.2026.1872725 (PMC13481716; doi:10.3389/finsc.2026.1872725)
Supplement: Supplementary file 1 [file Supplementaryfile1.docx]

Evaluation of CPP and LDH Nanocarriers for dsRNA efficiency in *Brassicogethes aeneus*

Triin Kallavus^1,2*^, Ly Porosk^3^, Liina Soonvald^1^, Apurva Vikas Sabnis^1,2^, Silva Vilumets^1^, Riina Kaasik^1^, Jonathan Willow^4^, Clauvis Nji Tizi Taning^2^, Kristof De Schutter^2^, Margus Pooga^3^, Eve Veromann^1^

**Supplementary Materials:**

**SI Table 1.** Green fluorescent protein (*gfp*) and *Brassicogethes aeneus* coatomer subunit alpha (*αCOP*) regions used in studies.

**SI Table 2.** Primers used in these studies.

**SI Figure 1.** Clay nanocarrier complexation with dsRNA 300 ng/µL in different ratios (0.1-1.2%), based on dry mass). dsRNA integrity was evaluated by agarose gel (1%) electrophoresis to determine whether dsRNA was intact or degraded. A DNA ladder (Thermo Scientific™ MassRuler™) was used only as a qualitative size reference, as exact fragment sizing was not the aim of the analysis.

**SI Figure 2.** **SI Figure 2.** Clay nanocarrier functional assays: (A) Zeta potential of pre-formed clay–dsRNA complexes, with free clay and free NA as controls. B) Accessibility of nucleic acid (NA) after interaction with clay particles was assessed using a fluorescent intercalating dye–based assay. Clay–NA mixtures (1:1 ratio) were prepared at final clay concentrations of 0.4%, 0.8%, and 1.2%. After 30 min incubation, samples were diluted to yield 33.3 ng NA per 10 µL and transferred to a black 96-well plate containing 90 µL of detection dye. Free dsRNA and blank samples served as 100% and 0% accessibility controls, respectively. (C) Increased NA accessibility was observed after incubation of loaded particles with the polyanion heparin, assessed using the same fluorescent assay. Statistical analysis was performed using one-way ANOVA with Dunnett’s multiple comparisons. In (B), results were compared to free NA; in (C), to particles treated with water instead of heparin.

**SI Figure 3.** Effect of clay nanocarrier on dsRNA stability as analyzed by electrophoretic mobility assay. Complexes were formed between dsGFP and the clay nanocarrier at ratio 1.2% and 0.8% (based on dry mass) and incubated in *Brassicogethes aeneus* gut extract for 0, 15, 30 and 60 minutes. dsRNA integrity was evaluated by agarose gel (1%) electrophoresis to determine whether dsRNA was intact or degraded. A DNA ladder (Thermo Scientific™ MassRuler™) was used only as a qualitative size reference, as exact fragment sizing was not the aim of the analysis. The results indicate that clay nanocarrier MgAl LDH was able to form stable complexes with dsRNA and provided partial protection against degradation under gut-like conditions.

**SI Figure 4.** Functional performance and physicochemical characterization of PF14 peptide nanocarriers: (A) RNA binding efficiency of PF14–dsRNA complexes was determined using a fluorescent nucleic acid (NA) intercalating dye (RiboGreen) assay. Complexes were prepared at charge ratios (CR) 1–4 and incubated for 30 minutes at room temperature. The fluorescence signal, inversely proportional to RNA binding, was normalized to free dsRNA (100% accessible). NP is nanoparticle (B) Zeta potential was determined for the same complexes to evaluate surface charge characteristics. Free PF14 and dsRNA served as controls. (C) Hydrodynamic diameter of PF14–dsRNA complexes at CR1–CR4 was measured by dynamic light scattering (DLS) to assess particle size distribution and colloidal stabilityNP I nanoparticle. (D) Heparin displacement assay was performed to assess complex stability and interaction strength. Complexes were incubated with the polyanion heparin, and RNA accessibility was quantified using the fluorescent intercalating dye–based method. Data represent mean ± SD of three independent experiments. Statistical analysis was performed using one-way ANOVA with Dunnett’s multiple comparisons, comparing (A) against free dsRNA and (D) against complexes treated with water instead of heparin.

**SI Figure 5. .** Effect of PF14 peptide nanocarrier on dsRNA stability as analyzed by electrophoretic mobility assay. Complexes were formed between dsGFP and the peptide nanocarrier PF14 at a CR 2 and 3 of and incubated in *Brassicogethes aeneus* gut extract for 0, 15, 30, 60 and 120 minutes. dsRNA integrity was evaluated by agarose gel (1%) electrophoresis to determine whether dsRNA was intact or degraded. A DNA ladder (Thermo Scientific™ MassRuler™) was used only as a qualitative size reference, as exact fragment sizing was not the aim of the analysis. The results indicate that PF14 was able to form stable complexes with dsRNA and provided partial protection against degradation under gut-like conditions.

**SI Table 1.** Green fluorescent protein (*gfp*) and *Brassicogethes aeneus* coatomer subunit alpha (*αCOP*) regions used in studies.

| Gene | Name | Region length (bp) for dsRNA synthesis | Sequence of gene region |
| --- | --- | --- | --- |
| *αCOP* | *coatomer subunit alpha* | 213 | AAGATGCACCACATAATGGACATTTTTCTTCAGGTTTTCCTCTGTAAATGGGGGTGTATGAATAACCACAAATCGAAAATGGATTATGTTCATCGTAATTTAATTGAAGTTCATCTGTTAAATTAGAGTCACAGGCCTGTAAAATTTTTCTTGCCTGCTGAGCCACCTCAGGACGTGGGCCCAATTCCAATAGCCTCTTGGCCAGCGAAGACG |
| *gfp* | *green fluorescent protein* | 455 | TACGGCGTGCAGTGCTTCAGCCGCTACCCCGACCACATGAAGCAGCACGACTTCTTCAAGTCCGCCATGCCCGAAGGCTACGTCCAGGAGCGCACCATCTTCTTCAAGGACGACGGCAACTACAAGACCCGCGCCGAGGTGAAGTTCGAGGGCGACACCCTGGTGAACCGCATCGAGCTGAAGGGCATCGACTTCAAGGAGGACGGCAACATCCTGGGGCACAAGCTGGAGTACAACTACAACAGCCACAACGTCTATATCATGGCCGACAAGCAGAAGAACGGCATCAAGGTGAACTTCAAGATCCGCCACAACATCGAGGACGGCAGCGTGCAGCTCGCCGACCACTACCAGCAGAACACCCCCATCGGCGACGGCCCCGTGCTGCTGCCCGACAACCACTACCTGAGCACCCAGTCCGCCCTGAGCAAAGACCCCAACGAGAAGCGCGATCA |

**SI Table 2.** Primers used in these studies.

| Gene | Name | Forward (5' to 3') | Reverse (5' to 3') |
| --- | --- | --- | --- |
| *gfp* | green fluorescent protein | CACATGAAGCAGCACGACTT | TGCTCAGGTAGTGGTTGTCG |
| *αCOP* | coatomer subunit alpha | CCAACTTGGTCATTTAACAATCTGG | CCCTTCCTTCATCATGGACA |
| *rps3* | ribosomal protein S3 | CCAACGCGTACCGAAATTAT | GAGTTTTCGGGGAAGTTGAA |
| *actine* | actin | TCACGGACAATTTCCCTTTC | TATCCTCCGTTTGGACTTGG |
|  |  |  |  |

**SI Figure 1.** Clay nanocarrier complexation with dsRNA 300 ng/µL in different ratios (0.1-1.2%), based on dry mass). dsRNA integrity was evaluated by agarose gel (1%) electrophoresis to determine whether dsRNA was intact or degraded. A DNA ladder (Thermo Scientific™ MassRuler™) was used only as a qualitative size reference, as exact fragment sizing was not the aim of the analysis.

**SI Figure 2.** Clay nanocarrier functional assays: (A) Zeta potential of pre-formed clay–dsRNA complexes, with free clay and free NA as controls. B) Accessibility of nucleic acid (NA) after interaction with clay particles was assessed using a fluorescent intercalating dye–based assay. Clay–NA mixtures (1:1 ratio) were prepared at final clay concentrations of 0.4%, 0.8%, and 1.2%. After 30 min incubation, samples were diluted to yield 33.3 ng NA per 10 µL and transferred to a black 96-well plate containing 90 µL of detection dye. Free dsRNA and blank samples served as 100% and 0% accessibility controls, respectively. (C) Increased NA accessibility was observed after incubation of loaded particles with the polyanion heparin, assessed using the same fluorescent assay. Statistical analysis was performed using one-way ANOVA with Dunnett’s multiple comparisons. In (B), results were compared to free NA; in (C), to particles treated with water instead of heparin.

**SI Figure 3.** Effect of clay nanocarrier on dsRNA stability as analyzed by electrophoretic mobility assay. Complexes were formed between dsGFP and the clay nanocarrier at ratio 1.2% and 0.8% (based on dry mass) and incubated in *Brassicogethes aeneus* gut extract for 0, 15, 30 and 60 minutes. dsRNA integrity was evaluated by agarose gel (1%) electrophoresis to determine whether dsRNA was intact or degraded. A DNA ladder (Thermo Scientific™ MassRuler™) was used only as a qualitative size reference, as exact fragment sizing was not the aim of the analysis. The results indicate that clay nanocarrier MgAl LDH was able to form stable complexes with dsRNA and provided partial protection against degradation under gut-like conditions.

**SI Figure 4.** Functional performance and physicochemical characterization of PF14 peptide nanocarriers: (A) RNA binding efficiency of PF14–dsRNA complexes was determined using a fluorescent nucleic acid (NA) intercalating dye (RiboGreen) assay. Complexes were prepared at charge ratios (CR) 1–4 and incubated for 30 minutes at room temperature. The fluorescence signal, inversely proportional to RNA binding, was normalized to free dsRNA (100% accessible). NP is nanoparticle (B) Zeta potential was determined for the same complexes to evaluate surface charge characteristics. Free PF14 and dsRNA served as controls. (C) Hydrodynamic diameter of PF14–dsRNA complexes at CR1–CR4 was measured by dynamic light scattering (DLS) to assess particle size distribution and colloidal stabilityNP I nanoparticle. (D) Heparin displacement assay was performed to assess complex stability and interaction strength. Complexes were incubated with the polyanion heparin, and RNA accessibility was quantified using the fluorescent intercalating dye–based method. Data represent mean ± SD of three independent experiments. Statistical analysis was performed using one-way ANOVA with Dunnett’s multiple comparisons, comparing (A) against free dsRNA and (D) against complexes treated with water instead of heparin.

**SI Figure 5.** Effect of PF14 peptide nanocarrier on dsRNA stability as analyzed by electrophoretic mobility assay. Complexes were formed between dsGFP and the peptide nanocarrier PF14 at a CR 2 and 3 of and incubated in *Brassicogethes aeneus* gut extract for 0, 15, 30, 60 and 120 minutes. dsRNA integrity was evaluated by agarose gel (1%) electrophoresis to determine whether dsRNA was intact or degraded. A DNA ladder (Thermo Scientific™ MassRuler™) was used only as a qualitative size reference, as exact fragment sizing was not the aim of the analysis. The results indicate that PF14 was able to form stable complexes with dsRNA and provided partial protection against degradation under gut-like conditions.
